# Supplementary material for: Aspergillus Enhances Eosinophil and Neutrophil Extracellular DNA Trap Formation in Chronic Rhinosinusitis
Source: Int J Mol Sci. 2023 Dec 8;24(24):17264. doi: 10.3390/ijms242417264 (PMC10744233; doi:10.3390/ijms242417264)
Supplement: Supplementary file 1 [file ijms-24-17264-s001.zip › ijms-2706063-supplementary.pdf]

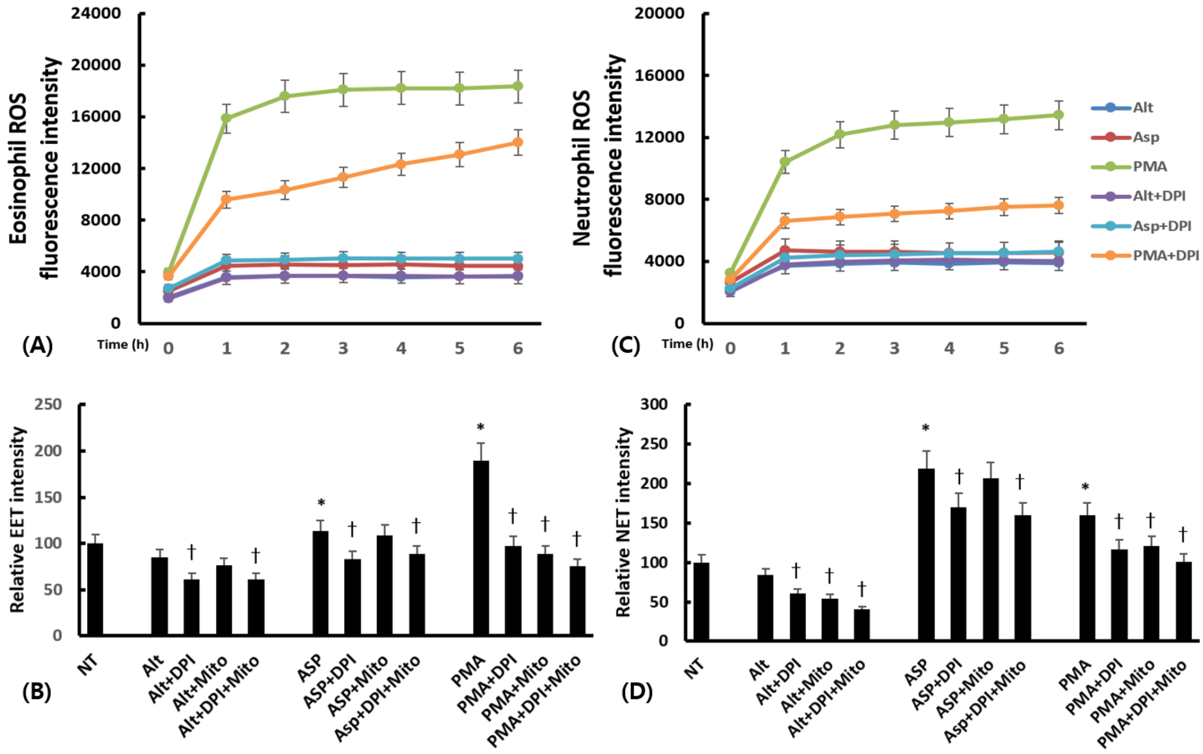

Figure S1. The effect of reactive oxygen species (ROS) on EET or NET formation in non-eosinophilic chronic rhinosinusitis by fungi. *Alternaria alternata* (Alt) and *Aspergillus fumigatus* (Asp) enhanced intracellular ROS production in a time-dependent manner (A and B). Nicotinamide adenine dinucleotide phosphate-oxidase inhibitor, diphenyleneiodonium (DPI), significantly suppressed both *Aspegillus*-induced EET and NET formation (C and D). PMA was used as a positive control. NT: not-treated, Mito: mitochondrial ROS inhibitor, MitoTempo, * $p < 0.05$ compared with NT, † $p < 0.05$ compared with Alt, n = 5.

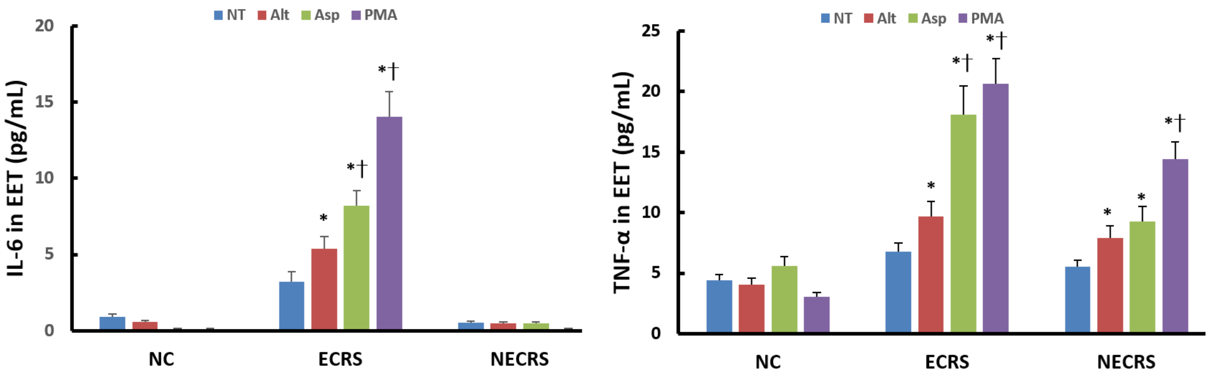

Figure S2. Interleukine-6 (IL-6) and tumor necrosis factor (TNF)-*α* concentration in eosinophilic extracellular traps (EETs). *Alternaria alternata* (Alt) and *Aspergillus fumigatus* (Asp) strongly induced IL-6 production from eosinophils in eosinophilic chronic rhinosinusitis (ECR) and TNF-*α* production from eosinophils in ECRS and eosinophilic chronic rhinosinusitis (NECRS). *Aspergillus* significantly enhanced IL-6 and TNF-*α* production compared to *Alternaria*. PMA was used as a positive control. NC: normal control, NT: not-treated, * $p < 0.05$ compared with NC, † $p < 0.05$ compared with Alt, n = 5.
